# Stability of Sunscreens Containing CePO_4_: Proposal for a New Inorganic UV Filter

**DOI:** 10.3390/molecules19079907

**Published:** 2014-07-09

**Authors:** Vitor C. Seixas, Osvaldo A. Serra

**Affiliations:** Department of Chemistry, Faculty of Philosophy, Sciences and Languages of Ribeirão Preto, University of São Paulo, Ribeirão Preto, SP 14040-901, Brazil; E-Mail: vitorcseixas@yahoo.com.br

**Keywords:** sunscreens, inorganic UV filter, chemical stability, rheological study

## Abstract

Inorganic UV filters have become attractive because of their role in protecting the skin from the damage caused by continuous exposure to the sun. However, their large refractive index and high photocatalytic activity have led to the development of alternative inorganic materials such as CePO_4_ for application as UV filters. This compound leaves a low amount of white residue on the skin and is highly stable. The aim of this study was to evaluate the physical and chemical stability of a cosmetic formulation containing ordinary organic UV filters combined with 5% CePO_4_, and, to compare it with other formulations containing the same vehicle with 5% TiO_2_ or ZnO as inorganic materials. The rheological behavior and chemical stability of the formulations containing these different UV filters were investigated. Results showed that the formulation containing CePO_4_ is a promising innovative UV filter due to its low interaction with organic filters, which culminates in longer shelf life when compared with traditional formulations containing ZnO or TiO_2_ filters. Moreover, the recognized ability of CePO_4_ to leave a low amount of white residue on the skin combined with great stability, suggests that CePO_4_ can be used as inorganic filter in high concentrations, affording formulations with high SPF values.

## 1. Introduction

Ultraviolet (UV) filters or sunscreens are widely used to protect the skin from UV radiation thus minimizing various detrimental effects including sunburn, photo-ageing processes, immunosuppression and, skin cancer. They consist of topical use formulations containing substances capable of either absorbing, reflecting or dispersing UV radiation. An ideal UV filter must present a high extent of skin substantivity with the minimum transdermal penetration into the systemic circulation. However, several studies have demonstrated that certain UV filters can penetrate the body via topical application [1,2,3].

In this context, inorganic UV filters such as titanium dioxide (TiO_2_) and zinc oxide (ZnO) have been introduced as supposedly safer alternatives to organic ones because they do not degrade when exposed to UV radiation, apart from not being able to penetrate the skin. Furthermore, they offer a wider spectrum and proven efficacy against UV-induced skin damage [4,5,6].

Despite their great efficacy in protecting the skin against UV damage, formulations containing opaque inorganic oxides show low cosmetic acceptability, reported by lower and insufficient application rates [4,6,7]. Their large refractive index may give the skin a peculiar white appearance, and their high photocatalytic activity facilitates the generation of reactive oxygen species, which can oxidize and degrade other substances in the formulation [8] resulting in instability and unsafe reactions.

Inorganic UV filters are inert materials constituted by particles that act as barriers to UVA and UVB radiations reflecting the light, as well as absorbing the UV radiation. The UV absorption results in electron mobility and transitions between certain electronic states forming excited species (electron/hole pairs). However, the higher transition mobility of these electrons may cause side reactions with the organic UV filters, excipients, or skin components, producing new species with unknown toxicological properties. In other words, this reaction could affect the stability, efficacy, and safety of the final formulation [8,9].

The scientific community has made many efforts to develop new and different inorganic UV filters to effectively block the solar radiation, which must be safe enough for use in cosmetic formulations, but at the same time capable to avoid undesirable characteristics such as the whitening effect, instability, and unpleasant sensations experienced during product application [10]. Therefore, inorganic particles based on rare earth (RE) elements have been proposed as inorganic UV filters in cosmetic formulations [8,9,10]. Particularly, cerium-based materials based have raised interest due to their abundance and several special properties. For example, cerium (IV) oxide has a low refractive index, is relatively transparent to visible light, possesses excellent UV absorption, and lower photocatalytic activity compared with TiO_2_ and ZnO [8,10].

Nevertheless, because of its catalytic activity for the oxidation of organic materials, CeO_2_ has been rarely used as sunscreen for commercial purposes. In the attempt to bypass this problem, the synthesis and application of CePO_4_ nanosized powders as UV filter have been accomplished [10,11]. CePO_4_ presents similar characteristics of CeO_2_ as well as reduced catalytic and photocatalytic activities, thus preventing the degradation of organic materials in cosmetic formulations [12].

During the development of cosmetic formulations, mainly of those containing inorganic UV filters, the stability and the physical characteristics must be considered and well evaluated to ensure their safety and efficacy during their whole shelf life. Besides the characteristics of the active substances, the physical and chemical properties of the vehicle, such as pH and water content, and the combination of active substances must also be considered, so that the stability of cosmetic formulations containing UV filters is guaranteed. The materials which compose the vehicle must be compatible with the selected active substances, in order to fulfill the indicated use of the product [13,14].

The physical stability of a cosmetic formulation can be evaluated by studying its rheological behavior under thermal stress. In this condition, prediction of instability processes is possible because some parameters such as viscosity, solubility, creaming facilitation, coalescence, melting of waxes, and hydration of polymers are altered [15]. Therefore, it is important to understand the rheological behavior during the formulation, handling, mixing, processing, transport, storage, and application of such formulations [16].

In this sense, High Pressure Liquid Chromatography (HPLC) measurements are often employed to obtain data concerning the chemical stability of the formulations during long periods of storage [16]. In order to predict the shelf life of the formulation, the kinetics of chemical degradation may be mathematically treated by using the Arrhenius equation [17].

Another important factor to take into account in the development of cosmetic formulations containing inorganic UV filters is the zeta potential values; it allows evaluating the behavior of the inorganic UV filters, since its intrinsic characteristics play an important role in the formulation stability. The zeta potential is a parameter that indicates the properties of the interface and of the solid solution near the interface [18,19]. The electrophoretic mobility of the dispersed particles in charged electric fields is usually used for zeta potential measurements. High values of zeta potential are important for the physical and chemical emulsion stability, since repulsive forces tend to avoid possible flocculation [18,19].

The present study aims to evaluate the physical and chemical stability of a cosmetic formulation containing ordinary organic UV filters combined with 5% CePO_4_, and, to compare it with other formulations containing the same vehicle and with 5% TiO_2_ or ZnO as inorganic materials. The main goal is to understand the behavior of CePO_4_ when applied in cosmetic formulations, its effect on the chemical stability of a cosmetic formulation, if it participates in side reactions along with organic UV filters and, its applicability as an inorganic UV filter.

## 2. Results and Discussion

In this study, CePO_4_ previously proposed as an innovative inorganic UV filter [10,11] was employed in association with traditional organic UV filters to evaluate its applicability. The formulation containing the new UV filter could culminate in a product with higher stability avoiding the undesirable whitening effect. To this end, the physical and chemical aspects of formulations containing organic UV filters supplemented with CePO_4_, TiO_2_, or ZnO were evaluated and compared.

**Figure 1:**
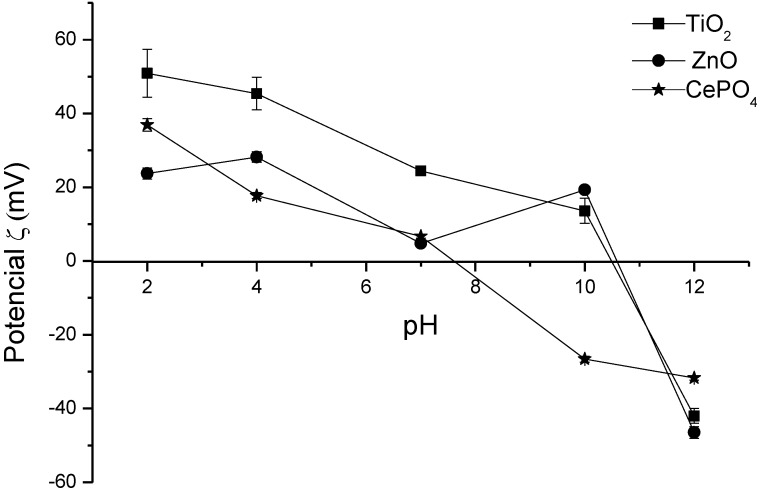
Zeta potential measurements of the inorganic UV filters.

Initially, the zeta potential measurement of the inorganic UV filters added to the formulations was accomplished. Since these inorganic materials are known to be insoluble in the formulation environment, they were suspended in the medium; this situation is related to the emulsion stability and may lead to phase separation, flocculation or coalescence [18,19,20]. High zeta potential values (in modulus) indicate greater electrostatic repulsion for the particles suggesting greater stability for the studied formulations [18,19,20]. The results demonstrate that ZnO, TiO_2_ and CePO_4_ possess sufficient charge for an adequate electrostatic repulsion, which should maintain the emulsion stability (Figure 1) [18,19,20].

### 2.1. Physical Stability

It is very important to evaluate the physical properties during the development of a topical formulation as these parameters indicate features related to spreadability, which is primordial when considering the consumer’s first contact with the formulation and it can determine and influence their choice of product. In addition, rheological characteristics also indicate changes and instability signals caused by the addition of some active substances [21].

Rheological measurements can be used to characterize cosmetic products such as creams, lotions, and gels. Those are plastic materials characterized by a non-Newtonian flow behavior. On the basis of the critical shear stress at the yield point, the emulsion type can be determined for creams as well as lotions [22]. Therefore, we developed an emulsion of oil/water (o/w) type and evaluated its physical changes as a function of time and under stress (37 °C and 45 °C) for the different formulations prepared in this study.

**Figure 2:**
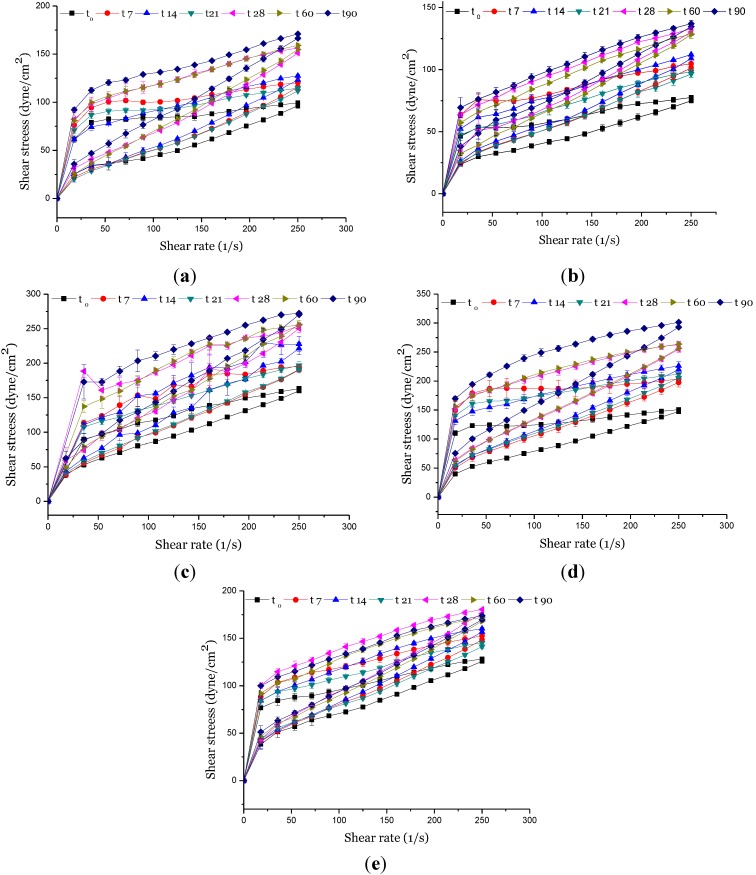
Rheograms of the (**a**) vehicle formulations; (**b**) formulation containing organic UV filters, and supplemented with (**c**) CePO_4_ or (**d**) TiO_2_ or (**e**) ZnO, when stored at 25 °C, in 0, 7, 14, 21, 28, 60 and 80 days after preparation.

**Figure 3:**
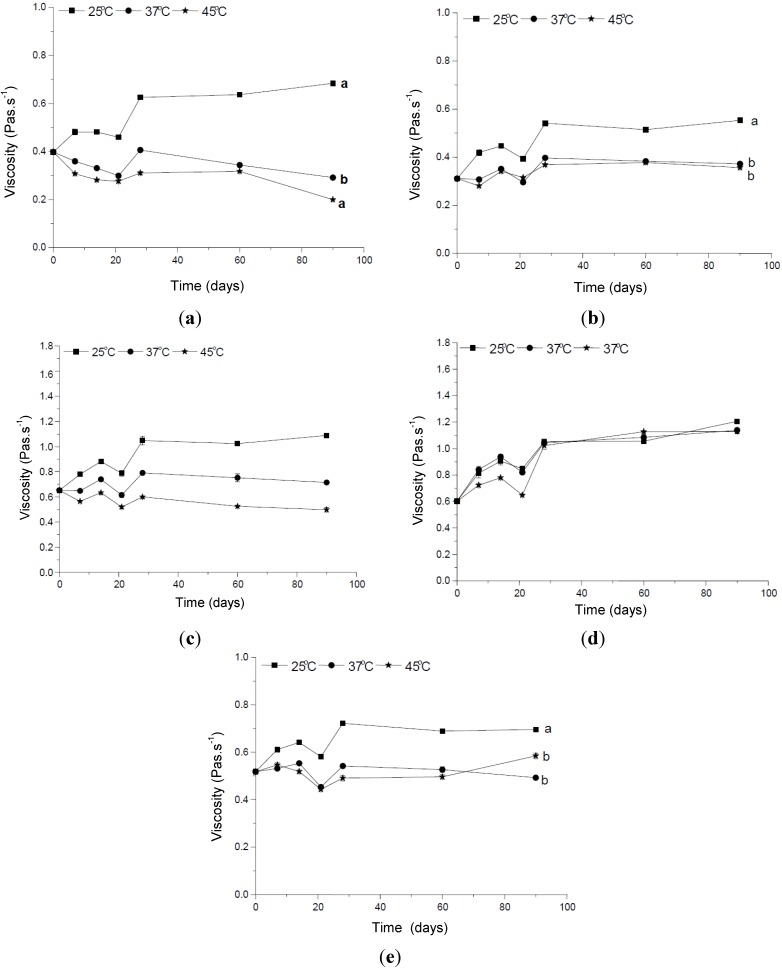
Changes in the viscosity *versus* storage times at different temperatures of the formulations: (**a**) vehicle; (**b**) containing the organic UV filters only, (**c**) supplemented with CePO_4_, (**d**) supplemented with TiO_2_, and (**e**) supplemented with ZnO.

**Figure 4:**
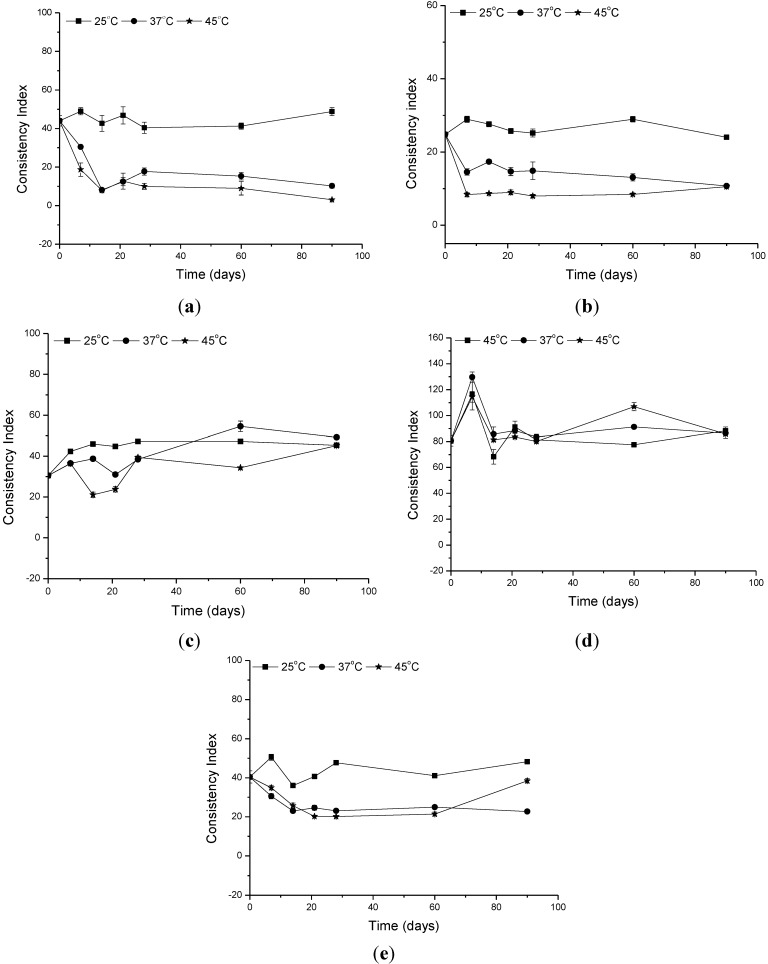
Changes in the consistency index of the (**a**) formulation vehicle (**b**) formulation containing the organic UV filters (**c**) formulation supplemented with CePO_4_ (**d**) formulation supplemented with TiO_2_; and (**e**) formulation supplemented with ZnO at different storage times and temperatures.

**Figure 5:**
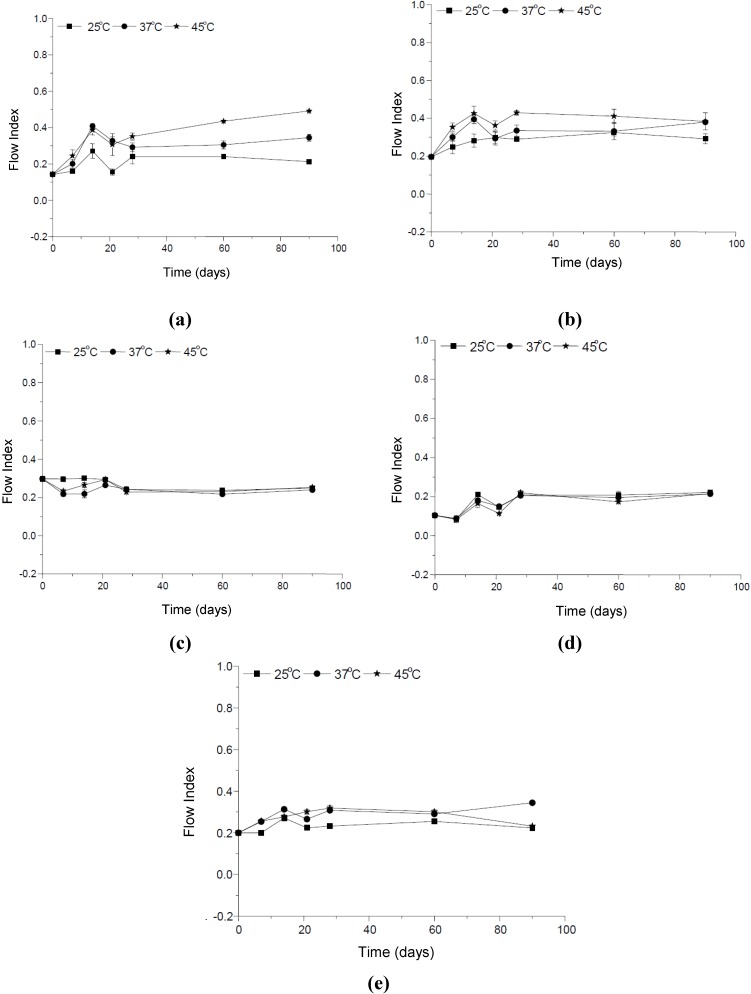
Changes in the flow index *versus* storage time at different temperatures (**a**) formulation vehicle; (**b**) formulation containing the organic UV filters; (**c**) formulation supplemented with CePO_4_; (**d**) formulation supplemented with TiO_2_; and (**e**) formulation supplemented with ZnO.

**Figure 6:**
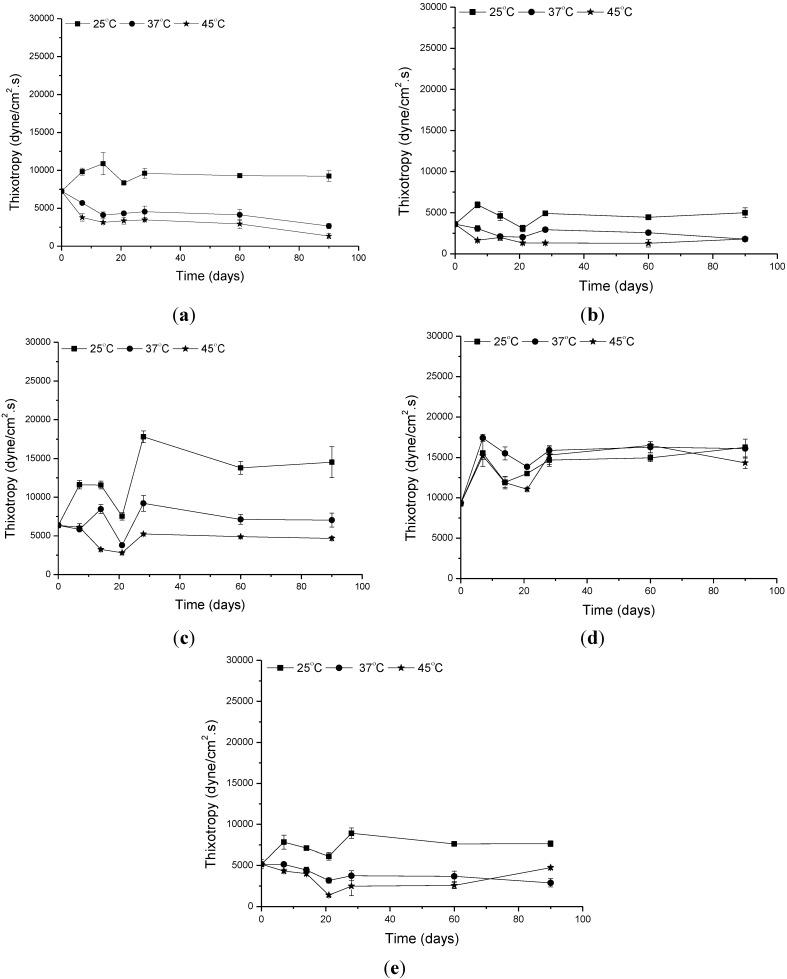
Changes in the thixotropy as a function of the time at different temperatures. (**a**) Formulation vehicle; (**b**) formulation containing the organic UV filters; (**c**) formulation supplemented with CePO_4_; (**d**) formulation supplemented with TiO_2_; and (**e**) formulation supplemented with ZnO.

**Figure 7:**
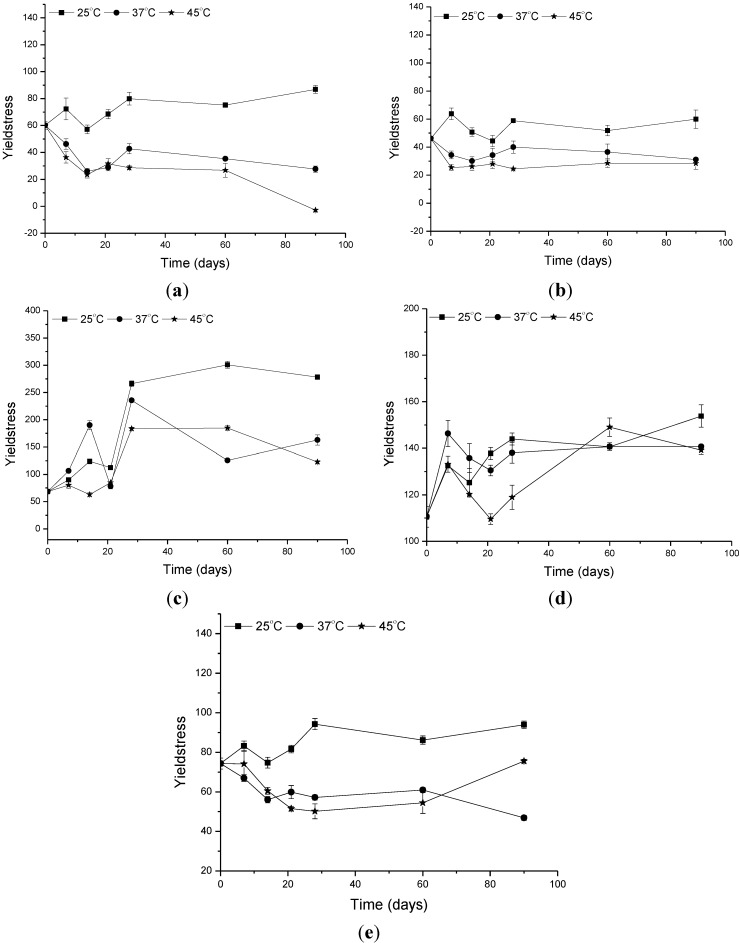
Changes in the yield stress of the (**a**) formulation vehicle (**b**), formulation containing the organic UV filters (**c**) formulation supplemented with CePO_4_ (**d**) formulation supplemented with TiO_2_ (**e**) formulation supplemented with ZnO in 0, 7, 14, 21 and 28 days after preparation, at 25 °C, 37 °C and 45 °C.

The rheological parameters showed that the addition of organic and inorganic UV filters to the vehicle formulation did not modify the properties of the initial formulation, since no peaks or other significant changes were detected in the rheogram curves (Figure 2). However, comparison between the formulations in terms of storage temperatures (25 °C, 37 °C and 45 °C), differences for some parameters such as apparent viscosity, consistency index, flow index, tixotrophy, and yield stress during the experimental period were observed, as described in Figure 2, Figure 3, Figure 4, Figure 5, Figure 6 and Figure 7.

A reduction in the apparent viscosity of the formulations supplemented with CePO_4_ or ZnO, stored at 37 °C and 45 °C was verified (Figure 3) and compared with the same formulations at 25 °C. These results suggest that the addition of these inorganic UV filters led to changes in the viscosity of the formulation over time. This is a common behavior of sunscreens containing a higher percentage of inorganic UV filters added directly to the emulsion vehicles as powders, which culminates in flocculation in a liquid [23]. However, these results cannot be considered as signs of instability because no peaks were observed during the time of study in any of the studied temperatures [24].

On the other hand, there was a significant increase in the apparent viscosity for the formulations supplemented with CePO_4_ and TiO_2_ stored at 25 °C. This is a desirable characteristic of a sunscreen formulation, since higher viscosity values can result in better photoprotection efficacy, as the consumer usually tends to apply a thicker layer of the product, culminating in a more effective film (higher sun protector factor—SPF) [25].

The consistency index is a measure of tenacity and is equivalent to the apparent viscosity at a shear rate of 1 s^−1^ [25]. The consistency indexes of the formulations are presented in Figure 4. A reduction in this parameter can be observed in the formulations containing ZnO, storage at 37 °C or 45 °C, compared with the same formulation at 25 °C. Once again, this result indicated that the addition of ZnO to the vehicle can alter the consistency of the formulation over time, which is in accordance with the data obtained for the viscosity parameter. This result was not observed for the formulations containing CePO_4_ and TiO_2_ stored at the same temperatures and for the same amount of time.

The flow index is a measure of the deviation of a system from the Newtonian behavior (*n* = 1). A value of *n* < 1 indicates pseudoplastic flow or shear thinning, whereas *n* > 1 represents dilatants or shear thickening flow [13,14,15,24,25]. The studied formulations furnished flow indexes varying from 0.0830 to 0.4915 evidencing a pseudoplastic flow behavior, as illustrated in Figure 4. Generally, the thicker the base, the lower the flow index, so the product with a high consistency index is less spreadable [25].

Pseudoplasticity is a desirable rheological property in cosmetic formulations. It improves application and spreading, thus providing a pleasant sensory feeling. Sunscreen formulations with a pseudoplastic flow produce a coherent protective film that covers the skin surface with evenly distributed UV filters, which is important for a higher SPF [13,15,24]. Newtonian materials do not behave in this way, because they run very quickly when spread on the skin, hence reducing the protective factor of the film [24]. The pseudoplastic material, on the other hand, can break down for easy spreading, and the applied film can instantaneously gain viscosity so as resist running [13,24].

The flow index of the formulations containing ZnO and stored at 37 °C and 45 °C was lower when compared with the same formulation stored at 25 °C. This is an evidence that this inorganic UV filter changes the spreadability of the vehicle formulation.

Thixotropy, or time-dependent flow, occurs because the product requires a finite time to rebuild its original structure, which breaks down during continuous shear measurements [21,25,26]. It is noteworthy that thixotropy is a desirable characteristic in cosmetic preparations, in terms of both engineering design and consumer application, since an initially thick product can be delivered as a thinner one, improving its spreadable property. Hysteresis areas (thixotropy), a pseudoplastic natural feature observed in the rheograms (Figure 2), were changed by addition of CePO_4_ and ZnO to the formulations stored at 37 °C and 45 °C (Figure 6C,E). These results suggest that the presence of these inorganic UV filters into vehicle negatively affects the film thus promoting changes in water resistance as well as in the SPF [24].

Another physical parameter of cosmetic formulations that must be evaluated is the yield stress. Substances with plastic flow properties present yield stress. The shear stress can be increased up to a specific value without any deformation taking place when the resistance is high. If the maximum value is exceeded the substance begins to flow because a sharp decrease in the viscosity takes place [13,14,22].

Dispersions with a high proportion of dispersed phase like emulsions usually exhibit plastic behavior because of the various interactions established between the dispersed particles. A solvation often sheath forms around the particles, immobilizing the external phase [13,14,22].

Whether a cream will have a good skin feel depends on several rheological factors. A high yield stress is desirable during removal of a cream from the packaging and at the start of application, but this should be quickly exceeded during the course of further application. Thus, a high yield stress means that the structural strength of the product is also high, which causes the product to adhere to the wall of the bottle. The force needed to cause the product to flow is so large that the product does not begin to flow under its own weight [22].

The critical shear stress of stable samples does not depend on time. For unstable samples, the apparent yield stress is shifted to higher values. The formulation containing ZnO had a significantly different critical shear stress parameter it was stored at 37 °C or 45 °C. This is yet another physical change happening in the vehicle as a result of the addition of this inorganic UV filter. Addition of TiO_2_ to the formulation increased the viscosity, consistency index and tixotrophy of the vehicle, which may be related to the solvation phenomenon [22,25]. The CePO_4_ also increased the viscosity, flow index, and yield stress parameters, which could be a sign of the stability of the formulation.

### 2.2. Chemical Stability

In this study, HPLC with UV detection technique was employed to verify the chemical stability of the produced formulations. This method is commonly used in the analysis of drugs and cosmetics [13,14,15], and it allows for the quantitative and qualitative assessment of organic UV filters [14,24]. Here, we will associate the stability of the formulation with the degradation of the organic UV filters, since these substances can undergo through oxidation processes. In this sense, we were able to evaluate the influence of the inorganic UV filters on the chemical stability of the formulations and thus on their shelf life.

**Figure 8:**
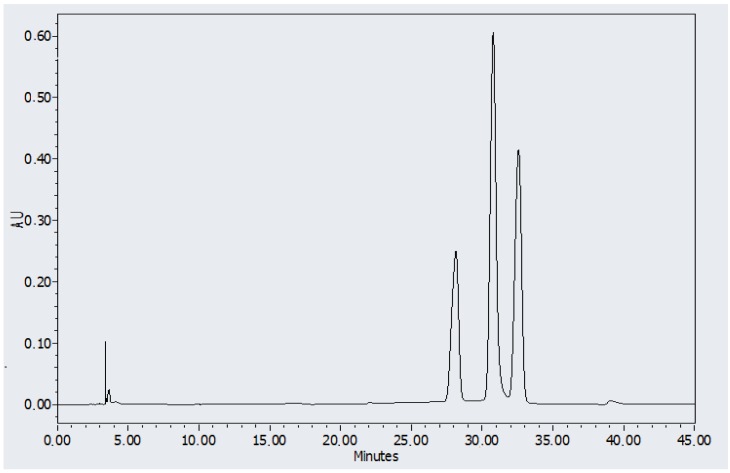
HPLC chromatogram of EEM, AV and OCT in a concentration of 100 μg/mL.

A representative HPLC chromatogram of the extracts of the organic UV filters ethylhexylmethoxycinnamate (EEM), octocrylene (OCT), and avobenzone (AVO) present in the formulation of the vehicle is given in Figure 8. It shows well-resolved EEM, AVO, and OCT peaks with retention times of about 28.14, 30.77, and 32.56 min, respectively, in a single and short easily reproducible analysis. The shelf life of EEM, AV, and OCT was calculated by using first-order chemical kinetics equations and 85% of the initial concentration was obtained as the remaining concentration.

**Figure 9:**
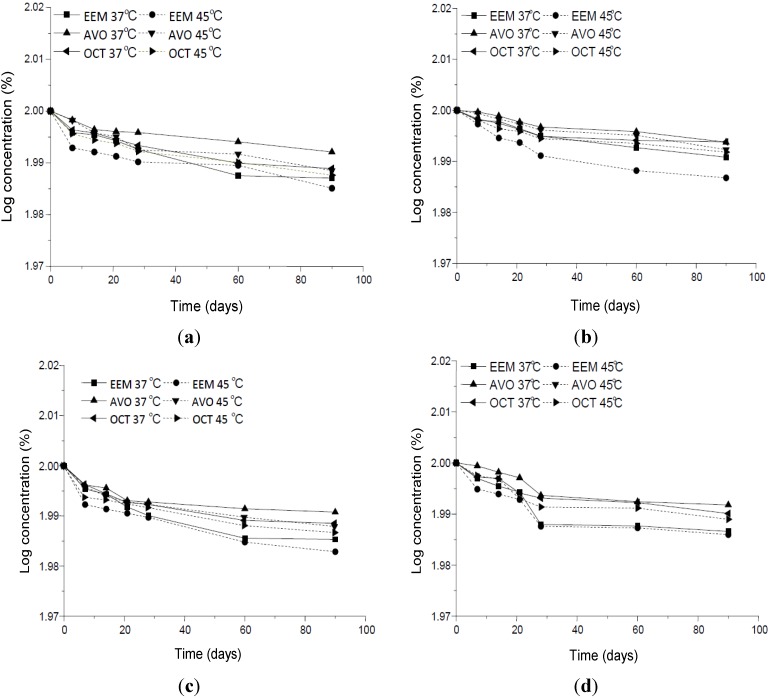
Quantification of EEM, (AVO) and (OCT), (**a**) alone or in combination with the inorganic UV filters (**b**) CePO_4_, (**c**) TiO_2_ and (**d**) ZnO, expressed as logs of concentration values over time formulations were, stored at 37 °C and 45 °C with 75% RH.

The different organic UV filters, alone or in combination with the inorganic UV filters, presented different degradation kinetics (Figure 9a–d). When these substances were combined with TiO_2_ and ZnO, their degradation rate was slightly higher than when they were alone. However, their association with CePO_4_ furnished slightly lower degradation rates, culminating in a longer shelf life (Table 1). The precision and accuracy/recovery of the method (intra- and inter-assay) are listed in Table 2.

**Table 1:** Shelf lives of the organic UV filters ethylhexyl methoxycinnamate (EEM), avobenzone (AVO) and octocrylene (OCT), alone or in combination with the inorganic UV filters ZnO, TiO_2_ and CePO_4_, stored at 45 °C and 37 °C, 75% RH.

Formulation	Shelf-Life (Days)		
	EEM	AVO	OCT
Organic UV filters	583	743	386
Organic UV filters + ZnO	409	1733	369
Organic UV filters + TiO_2_	378	346	410
Organic UV filters + CePO_4_	1435	1595	643

**Table 2:** Intra- and inter-day precision and accuracy/recovery (%) during the quantification of avobenzone, ethylhexyl methoxycinnamate and octocrylene extracted from experimental formulations.

UV Filter	Theoretical Concentration (μg·mL^−1^)	Intra-day			Inter-day		
		Obtained Concentration ± S.D. ^a^ (μg·mL^−1^)	Precision (R.S.D.%)	Accuracy/Recovery (%)	Obtained Concentration ± S.D. ^b^ (μg·mL^−1^)	Precision (R.S.D.%)	Accuracy/Recovery (%)
AVO	48	46.45± 0.48	1.03	96.76	46.40 ± 0.85	4.10	96.68
EEM	96	93.79 ± 1.10	1.17	98.01	94.96 ± 0.73	1.88	98.77
OCT	128	125.45 ± 1.47	1.17	97.70	126.63 ± 0.71	1.50	98.82

^a^ Standard deviation (intra day *n* = 5); ^b^ Standard deviation (inter day *n* = 17).

The shelf lives of EEM, AVO, and OCT in the different formulations are summarized in Table 2, in which it is possible to observe differences between the formulation containing the organic UV filter alone and in association with the different inorganic UV filters. The same results were obtained experimentally for the formulations maintained at room temperature (25 °C), which validates the accelerated method employed for determination of the chemical stability [13,14,15].

The organic UV filter that presented the highest degradation over time and under the stress conditions was octocrylene. Therefore, the shelf life determined for this substance was used to predict the shelf life of other formulations under study. Due to their high reactivity, UV filter compounds present low stability, so the development of stable sunscreens is still a challenge. In addition, the association of inorganic UV filters with organic ones has been associated with higher instability of the system, because the former can act as catalytic substances in some chemical reactions, resulting in even larger instability [8,9,27,28,29,30].

This information emphasizes the importance of assessing the chemical stability of a sunscreen, since instability is correlated with the efficacy and safety of the final product [13,14,15]. It was possible to observe a reduction in the shelf life of the formulations containing ZnO and TiO_2_ compared with the formulation constituted of the organic UV filters only (Table 2). This result can be related to the higher catalytic activity of these substances, which leads to the degradation of the organic UV filters [8,9,27,28,29,30]. In contrast, the shelf life obtained for the formulations containing CePO_4_ was higher than that of the formulation composed of organic substances only.

In general, our results suggest that CePO_4_ is a promising innovative inorganic UV filter that can be used in higher concentration for production of sunscreens with higher SPF values and longer shelf life.

## 3. Experimental Section

### 3.1. Formulations Studied

Formulations based on potassium cetyl phosphate (Table 3) were prepared in a Marconi^®^ MA1039 (Piracicaba, Brazil) stirrer at 650 rpm and supplemented with the organic UV filters ethylhexylmethoxycinnamate (EEM) 6%, octocrylene (OCT) 8%, and avobenzone (AVO) 3%, and with 5% CePO_4_ (synthesized as described by Lima *et al.—*Licensed for Silvestre Labs Brazil, Rio de Janeiro, Brazil) [11]. Formulations based on TiO_2_ (Nikkol, Tokyo, Japan) or ZnO (Vetec, Duque de Caxias, Brazil) were also prepared the same organic UV filters, for comparison purposes.

**Table 3:** Components of the formulation under study.

Components	(%, w/w)				
	Formulations				
	A	B	C	D	E
Potassium cetyl phosphate	3.00	3.00	3.00	3.00	3.00
Propylene glycol	2.50	2.50	2.50	2.50	2.50
Glycerin	2.50	2.50	2.50	2.50	2.50
PVP/Eicosene copolymer	2.00	2.00	2.00	2.00	2.00
Butylhydroxytoluene (BHT)	0.05	0.05	0.05	0.05	0.05
Disodium ethylenediamine tetraacetate (EDTA)	0.10	0.10	0.10	0.10	0.10
Capric caprylic triglyceride	1.00	1.00	1.00	1.00	1.00
Xanthan gum	0.30	0.30	0.30	0.30	0.30
Phenoxyethanol, methylparaben, ethylparaben, propylparaben, butylparaben, isobutylparaben	0.80	0.80	0.80	0.80	0.80
Cyclomethicone	2.00	2.00	2.00	2.00	2.00
Cyclomethicone (and) dimethicone crosspolymer	2.00	2.00	2.00	2.00	2.00
Ethylhexylmethoxycinnamate	-	6.00	6.00	6.00	6.00
Octocrylene	-	8.00	8.00	8.00	8.00
Avobenzone	-	3.00	3.00	3.00	3.00
CePO_4_	-	-	5.00	-	-
TiO_2_	-	-	-	5.00	-
ZnO	-	-	-	-	5.00
Distilled water	83.75	66.75	61.75	61.75	61.75

### 3.2. Stability Studies

Formulations containing the studied inorganic UV filters were stored in PVC pots (37 mm diameter × 29 mm depth), at 45 °C, 37 °C, and 25 °C and 75% relative humidity (RH) for up to 90 days. Rheological measurements sample collection, and quantification of the organic UV filters (EEM, OCT, and AVO) were accomplished in 7-day intervals for 28 days, and in days 60 and 90. All the quantifications were performed by a validated non-isocratic gradient HPLC method. To predict the formulation shelf life, the results were mathematically treated by using the Arrhenius equation, which characterizes the chemical stability of the formulations [13,14,15]. Considering that an increase in the storage temperature can decrease the chemical stability of a formulation [13,15], the organic UV filter quantifications also indicate the degradation kinetics. Thus, chemical stability should be calculated for a period of time during which formulations maintained at room temperature (25 °C) undergo a maximum of 15% loss in the concentration of their main components.

The results obtained for the formulations containing CePO_4_ were compared with those formulations consisting of the organic filters only and those formulations containing, TiO_2_ or ZnO, to evaluate possible different behaviors.

Zeta-potential measurements were also conducted in order to examine the behavior of the different UV inorganic filters present in the formulations, since the intrinsic characteristics of the inorganic component play an important role in the stability of the formulation. The measurements were carried out on a Zeta-Sizer 3000 HAS (Malvern, Westborough, MA, USA) equipment at pH values of 2, 4, 7, 10 and 12.

#### 3.2.1. Physical Stability

Physical stability was assessed by rheological determinations performed on a Brookfield rotational rheometer (Middleboro, MA, USA) model DV-III with a cone-plate configuration, connected to the Brookfield software program, Rheo 2000. Rheograms and viscosity measurements were accomplished at 25 °C and using CP 45 spindle. To obtain the ascendant curve, rotation speeds were progressively increased (0–250 rpm) and the procedure was repeated in reverse with gradually decreasing speeds (250–0 rpm), for the descendant segment. The rheograms were mathematically analyzed by the Ostwald Law, and values of apparent viscosity, flow index (related to the degree of sample pseudoplasticity), consistency index, thixotropy, and yield stress were obtained. The numeric integration of the rheogram curves and the data were analyzed using a non-parametric test, Kruskal Wallis, by means of Microcal Origin 8.0 software [13,24].

#### 3.2.2. Chemical Stability

The HPLC system consisted of a Waters Liquid Chromatograph model e2695 (Milford, MA, USA) fitted with a variable wavelength UV detector (UV Waters 2998) and connected to a personal computer. Empower 2, Waters was used as the integrator program. Chromatographic separations were performed on a Waters^®^ Sun Fire C18 column filled with 5 µm particles as the stationary phase. Gradient elution was conducted by starting a mobile phase composed of methanol (50%), isopropanol (20%), and water (30%) at a flow rate 0.8 mL·min^−1^, until a final composition consisting of acetonitrile (41%) isopropanol (33%), and water (26%) was obtained. The injection volume was 50 µL, and the analyses were carried out at λ = 359 nm for avobenzone, λ = 309 nm for ethylhexylmethoxycinnamate, and λ = 304 nm for octocrylene.

Formulation samples (40 mg) were added to a 25 mL glass beaker containing 10 mL of isopropanol and dissolved ultrasonically [16,17,18,27]. The solution was then diluted to 25 mL in a volumetric flask, filtered, and used for injection into the HPLC system. To quantify EEM, OCT, and AVO in the formulations, standard solutions were prepared on a daily bases and analyzed by HPLC in parallel to the samples. Peak-area ratios were employed for calculations. As for the precision assays, samples were analyzed five times, and the intra-assay relative standard deviation (R.S.D.) was determined. The inter-assay R.S.D. was obtained by analyzing the samples three times on five different days. The intra- and inter-assay accuracy/recovery were obtained by comparison between the theoretical concentrations of standard substances added to the analyte-free topical formulations and those obtained from the chromatographic analysis [13,14,15,24].

## 4. Conclusions

Stability studies emphasize the importance of evaluating the physical stability of sunscreens mainly when they contain inorganic UV filters added as powders and which may modify the structure of the vehicle.

CePO_4_ can be considered a promising innovative UV filter compared with the traditional inorganic ZnO and TiO_2_ filters due to its low interaction with organic filters, which results in longer shelf life, leading to more stable formulations for consumers. Moreover, knowing that CePO_4_ furnished lower amounts of white residue and high stability when applied to the skin, it can be suggested that it may be used in higher concentrations in the formulations, affording high SPF values. Finally, the results of this study can contribute to the subsequent development of more stable sunscreens containing CePO_4_ as inorganic UV filter, encouraging new studies regarding not only the application of CePO_4_ as UV filter but also developments on its chemical properties as UV filters, aiming to provide consumers with sunscreens displaying better quality and efficacy.
